# Approaches to health promotion in higher education: A scoping review (2014–2024)

**DOI:** 10.34172/hpp.025.44629

**Published:** 2025-11-04

**Authors:** Manesh Muraleedharan, Dwight Figueiredo, Rechel Shrisunder, Sammita Jadhav

**Affiliations:** Symbiosis Institute of Health Sciences, Symbiosis International University, Pune, India

**Keywords:** Adolescent health, College health promotion, Health education, Health promotion, Student health services

## Abstract

**Background:**

Health promotion within higher education institutions is gaining increasing global attention, particularly in response to the growing health challenges faced by adolescents and young adults. This demographic often experiences a convergence of health-related issues, including poor dietary practices, substance use, mental health disorders, and obesity. These issues frequently interact in complex ways, influencing long-term health trajectories. This scoping review aimed to examine the breadth of strategies and research concerning health-promoting initiatives across universities worldwide, while identifying existing gaps in the literature.

**Methods:**

Following the PRISMA-ScR guidelines, a systematic search was conducted for peer-reviewed articles published between 2014 and 2024 using the SCOPUS and Web of Science databases. After screening and eligibility assessment, 272 articles were included in the final review.

**Results:**

Thematically, 19% of studies focused on health-related policies and frameworks, 19% on mental health and wellness, 16% on technology-assisted interventions, 14% on physical activity, 7% on nutrition, 6% on behavioral habits, and others addressed curriculum restructuring.

**Conclusion:**

Findings reveal that most interventions remain in preliminary stages of implementation, with limited follow-up studies evaluating their effectiveness or cost-efficiency. There is a pressing need for robust, longitudinal research to assess outcomes, particularly in low- and middle-income countries. Furthermore, regional disparities—especially the limited representation from Sub-Saharan Africa—underscore the necessity for inclusive, globally coordinated research networks to foster equitable health promotion across diverse educational settings.

## Introduction

 Health promotion among universities is gaining momentum globally, particularly in light of concerns regarding the health and well-being of adolescents and young adults.^1^ Emerging challenges across the globe stem from changing lifestyles, dietary habits, sleep patterns, peer influence, social media, technological advancements, and transformations in academic environments. Additionally, the increasing threats posed by pandemics, conflicts, and economic fluctuations are significant contributors to adverse health outcomes during adolescence and early adulthood. While traditional concerns such as drug abuse, risk-taking behavior, and sexually transmitted diseases persist, newer issues have been added to the list, including lifestyle-related diseases and mental health issues linked to social media and associated crimes.^2,3^

 Health promotion is defined as “the process of enabling people to increase control over, and to improve, their health” (World Health Organization, Glossary of Health Promotion, 2021), and should be tailored to the unique environment and needs of each individual or community. Within university settings, health promotion encompasses strategies and actions aimed at creating supportive academic, social, and physical environments, encouraging healthy behaviors, and integrating health into institutional policies, curricula, and services.^3^ This includes addressing areas such as mental health, substance use, physical activity, nutrition, sexual health, and social well-being, while promoting health equity and student engagement. A settings-based approach is often employed, recognizing the university as a key environment for developing lifelong health-related skills and values, as well as for reducing health disparities among diverse student populations.

 Adolescence and young adulthood are critical periods for the development of social connections. Students in higher educational institutions frequently engage in socialization, explore diverse diets and activities, form relationships with the opposite gender, and participate in sexual activity. However, these behaviors also carry risks, including the early onset of non-communicable diseases, nutritional imbalances, sexually transmitted diseases, mental health challenges, and academic or peer pressure.^4^ To address these issues, various organizations, governments, academic bodies, and higher educational institutions are actively formulating policies and strategies aimed at implementing health promotion within higher education settings.^4,5^ Several universities are already engaged in enhancing their health-promoting strategies. Nevertheless, many regions, particularly low- and middle-income areas, remain underrepresented or inactive within the global network of health-promoting universities and higher educational institutions.^6^

 The International Health Promoting Universities and Colleges Network is guided by the Okanagan Charter.^7^ Established in June 2015, the Charter aims to enhance the culture of universities and colleges by promoting health and well-being. It provides a unified framework to guide institutions toward achieving optimal health and wellness outcomes.^7^ Although reviews on this topic exist, most have focused solely on studies explicitly mentioning or utilizing the term “health promotion.” The objective of this review is to explore the various approaches employed globally to promote health and well-being within higher educational institutions and to identify existing research gaps. To ensure a comprehensive review, we included all aspects of health promotion, including physical and mental health, health facility-based interventions, and health education-related research and reports from all global regions.

## Methods

**Figure 1:**
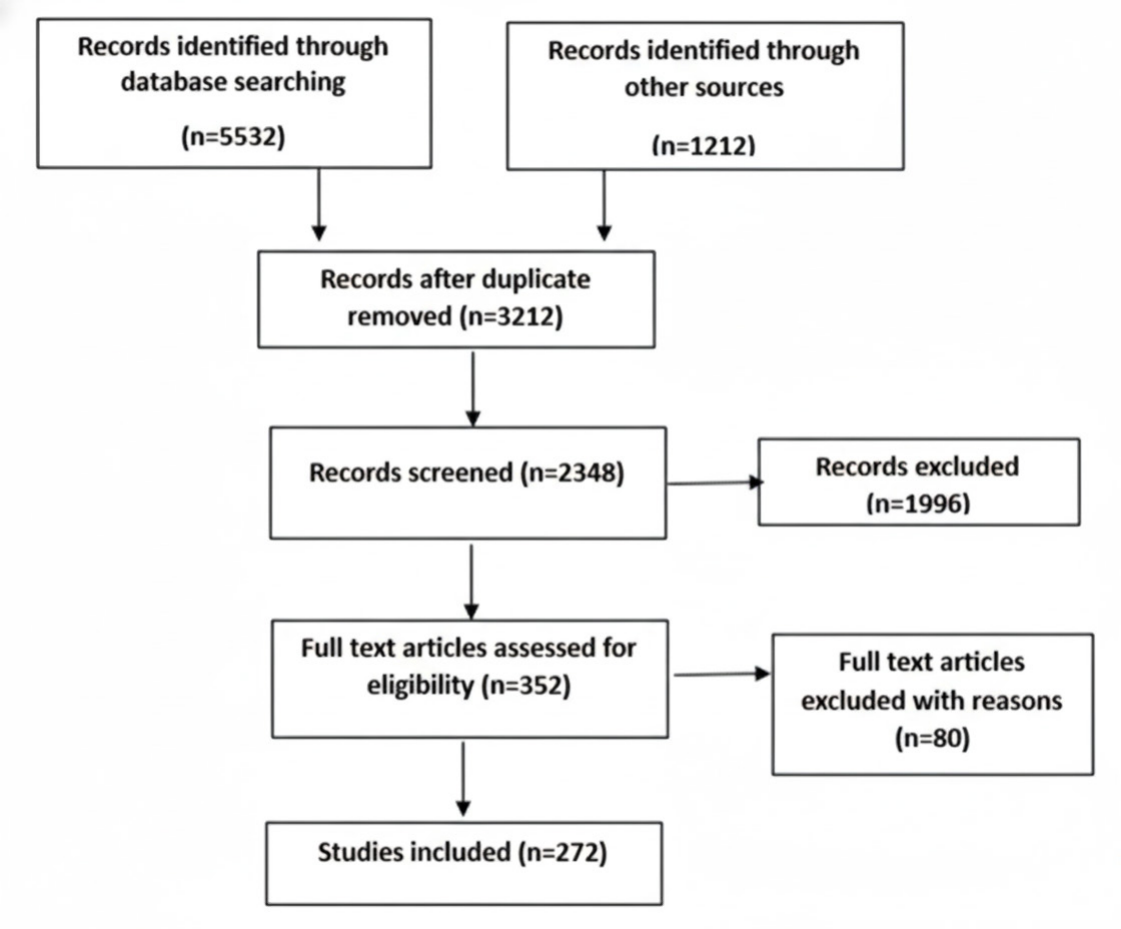
PRISMA flow chart of identifying and filtering articles for the review

 This scoping review included articles from 2014 to 2024 published in SCOPUS and Web of Science. A scoping review protocol was followed because of the evolutionary nature of the concept. The researchers followed the PRISMA guidelines-scoping review extension (PRISMA-ScR) (Figure 1).

###  Inclusion

 This study includes articles (research articles and reviews) published between 2014 and 2024. These articles included research related to health promotion in higher educational institutions, the effect of health promotion implemented in Higher Educational Institutions (HEIs), and policies related to health promotion in HEIs.

###  Exclusion

 Book chapters and conference proceedings were excluded. Also, surveys conducted to understand health awareness or health promotion-related awareness, COVID-19-related health promotion are excluded (our aim is to understand long term strategies and research related to health promotion among campuses). Articles related to health promotion that are only for employees of higher educational institutions are also excluded from our review.

###  Search terms

 MeSh terms, including combinations of “Health”, “Health promotion”, “Health education”, “College”, “Higher Educational Institution”, are appropriately used in search platforms (Table 1).

**Table 1:** Search strategy in databases using keywords and boolean operators.

**No.**	**Search terms and Boolean operators **
1	Health promotion AND Higher educational institution OR College OR University
2	Health education AND Higher educational institution OR College OR University
3	Health AND Higher educational institution OR College OR University

###  Analysis

 The downloaded articles were in Excel format, and four researchers screened them by reading the title to see the relevance in the first step. The second step involved screening the abstract, followed by the full text. Irrelevant articles were removed from the analysis after full-text reading, and the final list was made. Steps of inter-researcher reliability and discrepancy management:

1. Started with pilot screening of 5 articles by each researcher separately, made conclusions.
2. These conclusions were discussed to identify discrepancies and resolved through discussion and by aligning to the review objective.
3. Researchers in the next step divided the articles into half, a pair of researchers individually screened each half and decisions were made to include or exclude.
4. Cohen’s Kappa value was calculated based on agreement or disagreement of each researcher. The calculated values were 0.77 and 0.72. The set limit or agreement was 0.7 or above.

###  Process of coding and domain identification

 A standard common form was used to report the study findings, which included, authors, year of publication, type of article, interventional/ not, outcome (if applicable), codes, domain/ theme and major findings. The full text of each article was read by researchers and coding was done. Coding included initial inductive coding, followed by grouping of similar codes from each article. This helped us identify the domain of each article and major observations. In the last step, articles with similar domains are grouped to develop broader areas and discussion on the particular domain. In the final stage of domain identification, four authors jointly discussed each article and disparities were ironed out before deciding individual themes.

## Results

**Figure 2:**
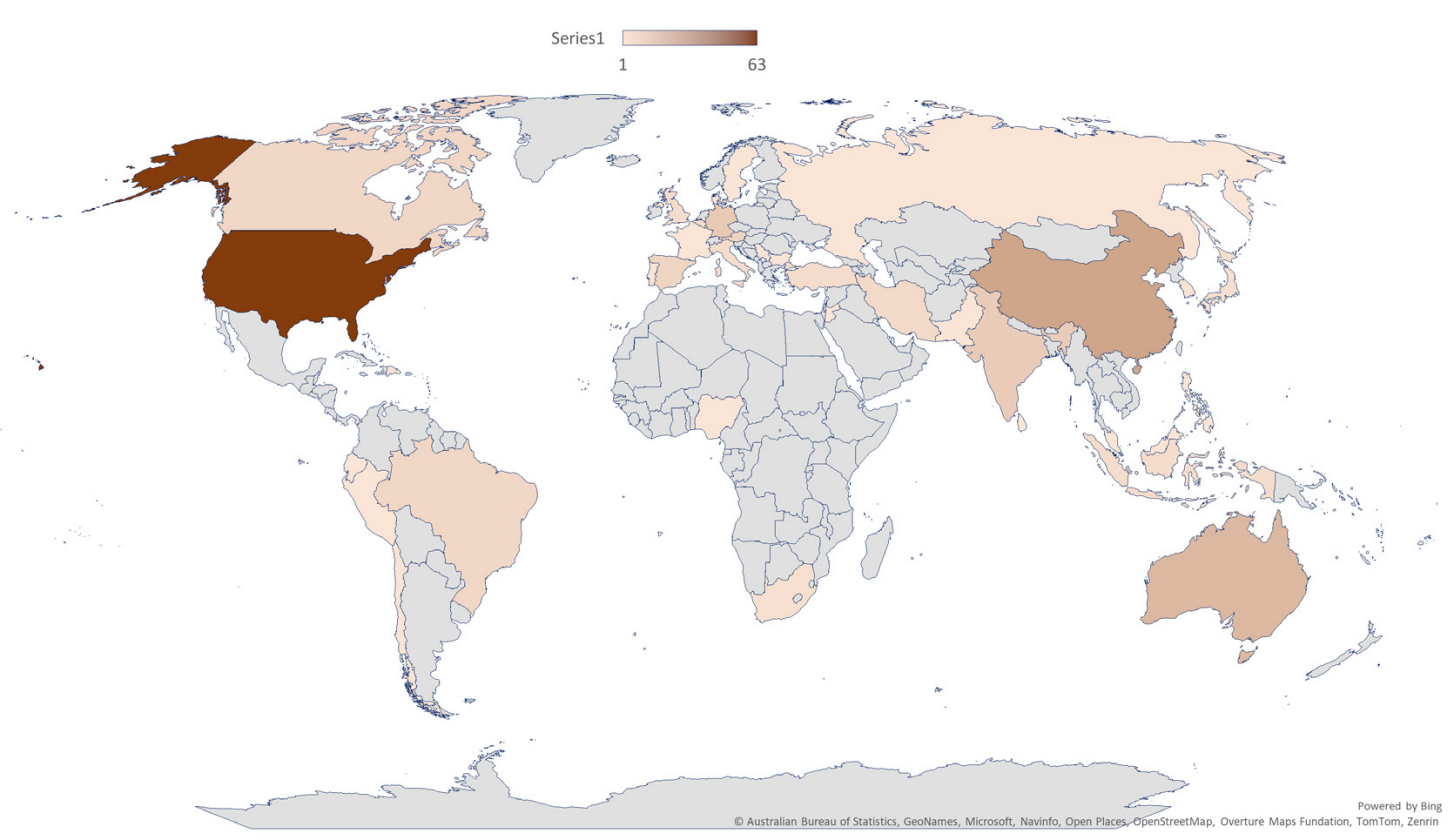
Geographical heat map of research contribution of various regions

 Geographical distribution of research related to this domain shows a clear dominance of the United States, which is 63 articles. Other countries like China (count-24), Australia (count-17), Germany (count-11), India (count-11), etc, are also significant contributors. However, the overall trend shows a lower contribution from Asian and Sub-Saharan African Regions (Figure 2).

**Figure 3:**
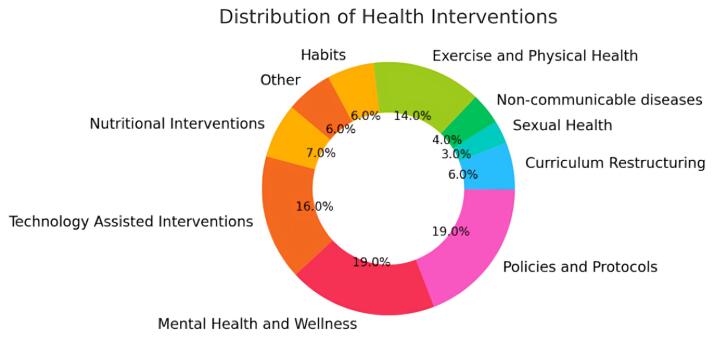
Distribution of domains identified from articles

 After filtering the articles using PRISMA guidelines for scoping review, we added 272 articles for the final review. The articles were further classified based on their major interventional or research domain (Figure 3). The majority of the articles were policy or framework related and also related to mental health or wellness interventions (19% each). Technology assisted interventions has a share of 16% among total included articles. Physical health or exercise-related interventions and research across the campuses were also dominant (14%). Other identified domains were nutritional interventions (7%), habits including smoking, drinking, binge eating and drinking etc (6%), sexual health (3%), curriculum restructuring (3%), self-screening and help-seeking (2%), facilities and environment (2%), oral health (1%) and non-communicable diseases (1%).

###  Policies and protocols

 With a significant share of 67 articles, this domain constituted the largest proportion in our review. The majority of these articles focused on policies related to the nationwide implementation of health-promoting university concepts, including guidelines for implementation, processes, and conceptual frameworks.^8^ However, reports concerning on-campus implementation and post-implementation outcome evaluations were not included in this category. Various aspects of health promotion and its requirements—such as physical health, mental health, health education, and health financing—were also addressed in several articles.^9^ Holistic health promotion concepts across institutions, wellness initiatives, habit-change interventions, and policies, including smoke-free campus policies, were frequently discussed.^10^ Additionally, student health screening and health education guidelines were examined in many studies. Health financing topics, including funding mechanisms for health promotion and the importance of student health insurance, were also explored in different articles.^11^ A few studies further discussed innovative frameworks and suggestions, such as community participation in campus health promotion and student-led health promotion initiatives.^9^

###  Mental health and wellness

 This area has the maximum share of articles (n = 68), discussing various interventions across campuses and also the effects of various interventions. Maximum share of interdisciplinary research areas is also identified in this domain, including improving physical health through mental health interventions, digital technology-enabled mental health promotion, dietary interventions combined with mental health promotion, etc.^12^ Various strategies and methods for mental health and wellness promotion across campuses include mindfulness practice, yoga, cognitive behavior therapy, meditation, etc.^13^ Sleep health promotion and weight loss through mental health interventions are also discussed in a few research articles.^14,15^ Positive psychology and suicide prevention activities were also implemented in some campuses. Some institutions also implemented animal-assisted wellness initiatives for students. Few universities promoted and studied peer-led mental health activities, wellness camps, mobile phone utilizing health-promoting initiatives like messaging or apps, online or web-based counselling and coaching, etc.^16^

###  Nutritional interventions

 Various strategies of diet-based interventions are implemented across campuses, including diet counselling, food labelling, healthy eating initiatives through campus eateries, promotion of dietary diversity, cooking training, etc.^17^ Few articles are interdisciplinary in this domain, which include online dietary education and web-based healthy eating courses for students.^18^ Unique initiatives in this domain are health promotion by providing healthy food in campuses, removing junk food from canteens or vending machines, and food labelling in campus canteens to easily identify healthy food and food-related information.^19,20^ Dietary education includes education regarding the optimum amount of nutrients, and some articles specifically describe dietary education for fruit and vegetable intake, water intake, dairy intake, etc.^21^ Promotion of cooking, especially cooking healthy food, has been discussed in two articles, including cooking competitions and promoting cooking through campus-led training.^19^

###  Technology assisted interventions

 A major share of articles were related to this domain (n = 56). Major initiatives include mobile-based interventions, AI-enabled interventions, and web-based interventions. Mobile-based interventions are the most common strategy, and articles discussed various health messaging interventions, WhatsApp-based health education, apps for tracking physical activity and weight loss, etc.^22^ Mobile applications to promote wellness and mental health are discussed in two articles. Advanced strategies like chatbot-enabled wellness programs, using virtual reality to promote mental health, audio-visual aided health education strategies, and using big data to develop interventions, were also discussed in various articles.^23^ However, more than half of the articles in this domain were related to various mobile-based interventions. Although various areas of health and wellness were covered by researchers, the majority of them were related to mental health or physical activity and exercise.^24^

###  Exercise and physical health

 Various strategies related to this domain include campus-led exercise initiatives, education related to physical activity, tracking of physical activity, specific trials like incentivising physical activity, and technology-enabled exercise and physical activity initiatives.^25^ Interdisciplinary areas of exercise and physical health include mobile-based applications for exercise and physical health education, as well as exercise or physical health associated with mental health.^25^ Promoting walking on campus, including peer-led walking initiatives and infrastructure reforms and utilisation, are also some strategies discussed in articles. Utilisation of the stairs and walkways of the campus environment is discussed in some articles.^26,27^ Inclusion of physical training or exercise as part of the curriculum is also discussed in some articles.^28^

###  Self-screening and help seeking

 Only a few [n = 6] articles were related to this domain. These articles were related to health education among students to identify disease symptoms or signs and to empower them to seek treatment.^29,30^ Various aspects covered were self-breast examination, menstrual hygiene, safe sex practices, tuberculosis prevention, symptoms, and treatment options. One article is related to a mobile-based messaging intervention to improve self-breast examination and breast care.^29,30^

###  Habits

 A significant number [n = 22] of articles were related to this domain. The majority of the articles were related to various interventions to stop smoking, the consumption of alcohol, or drug abuse.^31^ Various mobile phone-enabled interventions, like messaging or tracking of smoking or drinking to quit habits, have been discussed in many articles. Online training and tracking were also discussed in articles. Peer lead initiatives, awareness campaigns, poster presentations, and skills training among students were also implemented by some higher educational institutions.^32^ Two articles were related to training and promoting hand hygiene across campuses.^33^

###  Facilities and environment

 There were six articles related to this domain. Most of them were associated with the campus infrastructure to promote physical activities, especially walking.^34^ Natural and urban designs for walkways and redesigning the external environment of the classroom, to promote walking, were also discussed. Building specific health-promoting areas like fitness facilities, staircases, and healthy eateries was also discussed in a few articles.^35^

###  Oral health

 Only two articles were related to this domain. However, in some articles that discussed the overall health promotion and screening, oral health was also included. Specific articles related to oral health were related to oral hygiene and empowering students to seek oral health care, and also the inclusion of oral health screening across campuses.

###  Non-communicable diseases (NCDs)

 Few articles were associated with interventions for cardiovascular health. Some were related to obesity prevention and education. The majority of these were health education to prevent NCDs, and a few were about implementing regular health screening of students for NCDs.^36^

###  Curriculum restructuring

 Various campuses suggested or implemented health and wellness modules as part of their curriculum. The majority are physical activity or sports involvement requirements, mental health, or wellness education sessions. Some campuses discussed teaching or training in diet, mindfulness, yoga, meditation, or community engagement for health promotion. The majority of them are suggesting mandatory requirements of these courses to graduate, and some campuses consider them as elective or optional.^37^

## Discussion

 Rising trends of noncommunicable diseases, mental health issues, and habit-related diseases are concerning to the global community. Issues of lifestyle diseases, dietary imbalance, low mobility, and habits like smoking, drinking, and drug abuse are common among adolescents. Adolescents and young adults spend a significant amount of their time in higher educational institutions. To achieve the goal of the Sustainable Development Goals (SDGs), it is important to intervene with students across universities. This not only creates a healthy young generation but also a healthy promoting community across the globe. Various SDG goals are directly or indirectly linked to various health-promoting initiatives of higher educational institutions. These include links with SDG-1, 2, 3,4,5,6,8,9,10,11, and 12.

 After reading each article, we did an SDG mapping of the articles published and the topics (Table 2). Apart from SDG-3, linked to almost all the domains and interventions, SDG-4 and 5 were also linked to some areas in health promotion. Implementing health care aspects will improve the quality of education and the overall well-being of the young population and community. SDG-9 is also linked to much research and intervention in our analysis. Specifically, digital innovations for health promotion will engage students in various innovative platforms and ideas and equip them to build and use similar applications in the future. These innovative ideas will make them future-ready and industry-oriented (SDG-8). Infrastructure reforms required for health-promoting institutions will help students learn such innovations along with learning the need for health and well-being initiatives (SDG-9). It will further lead to sustainable solutions for a healthy future (SDG-11). Diet, physical activity, and infrastructure reforms will educate the students about the importance of responsible consumption and production (SDG-12). University students including adolescents and young adults are a major share of the population across the globe, especially Asian and Sub-Saharan African regions. To achieve SDG goals, it is important to identify health risks of this population and to implement interventions effectively.

**Table 2:** Domains identified and their link with various SDGs

**Domain**	**Priority 1**	**Priority 2**	**Priority 3**
Nutritional interventions	SDG-3	SDG-2	SDG-6
Technology assisted interventions	SDG-3	SDG-9	SDG-10
Mental health and wellness	SDG-3	SDG-5	SDG-10
Policies and protocols	SDG-3	SDG-4	SDG-9
Curriculum restructuring	SDG-4	SDG-3	SDG-8
Sexual health	SDG-3	SDG-5	SDG-10
Non-communicable diseases	SDG-3	SDG-10	SDG-11
Exercise and physical health	SDG-3	SDG-11	SDG-12
Self-screening and help seeking	SDG-3	SDG-5	SDG-11
Habits	SDG-3	SDG-4	SDG-11
Facilities and environment	SDG-9	SDG-3	SDG-10
Oral health	SDG-3	SDG-11	SDG-4
Other	SDG-3	SDG-9	SDG-5
2: Zero Hunger			
3: Good Health and Wellbeing			
4: Quality Education			
5: Gender Equality			
8: Decent Work and Economic Growth			
9: Industry, Innovation and Infrastructure			
10: Reduced Inequalities			
11: Sustainable Cities and Communities			
12: Responsible Consumption and Production			

Footnote: Color codes of each SDGs are used to represent the cells, based on UN listing.

 The Okanagan Charter is a global framework adopted in 2015 at an international conference in Kelowna, British Columbia (in the Okanagan region of Canada). It provides principles and calls to action for post-secondary institutions to integrate health into all aspects of campus culture and to lead health promotion action and collaboration. Major strategies suggested by Okangan Charter include 1) Embed health into all aspects of campus culture, including administration, operations, and academic mandates 2) Lead health promotion action and collaboration locally and globally.^2,3^ Also the charter suggests institutions that adopt the Okanagan Charter aim to build supportive environments that reduce stigma around mental health and improve access to services, promote physical activity and healthy eating through campus design and policies, ensure inclusive, safe, and equitable environments, use health data to inform campus-wide strategies, collaborate with students in co-creating health solutions. Major findings and interventions identified in this review are aligning with the suggestions of Okangan Charter including physical health, mental health, dietary interventions, technological innovations etc.^4^

 One of the major findings from the review is the higher number of policies, frameworks, and guidelines from various authorities, including national policies and guidelines for health promotion among campuses proposed by universities or educational institutions.^5,6^ This shows a growing trend of interest in health promotion among higher educational institutions. However, there is a noticeable disparity across regions in this aspect.^6^ Most of these policy or framework-related documents are from a few regions, like the USA and China, with a low share from most Asian and Sub-Saharan African regions. High population size and sluggish development in Asian and Sub-Saharan African regions, along with a significant number of universities and higher educational institutions, demand more attention across these regions.^7^

 The implementation of health promotion initiatives across low- and middle-income countries (LMICs) is often hindered by a range of structural and contextual challenges, including limited resources, large population sizes, and complex socio-political environments. These constraints impede the development and sustainability of comprehensive health promotion strategies, particularly within academic institutions. Addressing these barriers necessitates multifaceted approaches, such as increased scholarly engagement, curricular and educational reforms at both regional and global levels, and the allocation of dedicated funding by governmental authorities to support campus health initiatives. Furthermore, the active participation of non-governmental organizations (NGOs) can play a critical role in bridging gaps in service delivery and advocacy. Support from international bodies such as the United Nations (UN) and the World Health Organization (WHO) is equally essential in fostering global policy alignment, resource mobilization, and capacity building. Integrating campus health promotion into institutional accreditation frameworks and international ranking systems may also serve as a strategic lever to incentivize universities to prioritize health within their organizational agendas.

 Mental health issues are rising, specifically among youngsters, according to various organisations, including the WHO. One of the major areas of health promotion among university campuses is mental health and wellness promotion initiatives.^12,13^ Interdisciplinary areas like technology-enabled mental health and wellness initiatives, physical activities combined with mental health promotion, are also discussed by various universities and organisations. Most of these initiatives are in the initial stages of development or implementation. Hence, the acceptance or effectiveness of these interventions is less studied in the literature. Also, interventions like Yoga, meditation, and cognitive behavioral therapy need scientific proof from the student community to confirm their effectiveness.^14,15^ Similarly, various dietary interventions also require post-implementation evaluation. Only a few studies discussed the effect of such interventions, especially mobile phone or web-based nutritional interventions in the student population. One article discussed the inefficacy of milk vending in improving dairy and calcium intake among university students. Similarly, interventions like cooking training and promotion, and their relationship with health promotion among students, need evaluation.

 There is only minimal information available on curriculum reforms related to health promotion across campuses. The majority of these are related to health science, including medical, nursing, or paramedical curricula. Even though efforts are going on across all higher educational institutions to implement health promotion initiatives, data is scarce related to non-health science curricula.^28^ Another aspect is the importance of non-communicable diseases, and raising awareness and preventive aspects. Although exercise, diet, and other interventions are aimed at reducing the development of noncommunicable diseases, rising awareness and focused interventions for NCDs are also important.^36^

 Drinking, smoking, drug abuse, and other habit formations are also challenging problems among students and young adults. Various strategies to tackle this issue have been discussed in various articles. Counselling, health-promoting environments, like smoking-free campus initiatives and policies, are implemented in various campuses.^31,32^ However, similar to other interventions, effectiveness or data related to the large-scale implementation of these initiatives is still not adequately available. A very small number of articles are related to oral health promotion in our study. Oral health issues can affect the quality of life of individuals and can last a lifetime if left neglected. Screening, oral hygiene education, and oral health support are important in higher educational institutions.

 Various higher educational institutions are actively creating student-friendly and healthy environments across the globe. These include green spaces, spaces for physical activity and mental pleasure, healthy eating cafeterias, comfortable classrooms, and more.^34,35^ Environment-friendly and sustainable campuses are also gaining attention. However, such initiatives are required at a global level to share ideas and to understand the success factors from each other. Considering the number of initiatives across the globe, research related to this aspect is very less and the effectiveness of such initiatives. It is important to study various aspects of campus health promotion, including policies, implementation, and their effectiveness in various stages across the globe.

## Limitations of the review

 Only articles published in English were included in the final review, and conference proceedings as well as book chapters were excluded. As a result, there is a possibility that some relevant studies may have been omitted due to language and publication type restrictions. The majority of the articles were identified through SCOPUS and Web of Science databases, which may have led to the exclusion of certain forms of grey literature, such as dissertations, theses, and organizational reports. Additionally, health promotion and intervention studies specifically related to COVID-19 were excluded in order to maintain the long-term relevance of the review. However, this exclusion represents a further limitation, as it may omit context-specific insights relevant to recent global health developments.

## Conclusion and future directions

 Health promotion across university campuses is gaining momentum across the globe. However, most of these initiatives are in the policy-making stage or the initial stages of implementation. Most common interventions are mental health and wellness initiatives, followed by exercise and physical engagement, which are implemented or under the early stages of implementation in many campuses. The importance of diet during a younger age is growing research interest. Various effective strategies to promote healthy eating and their effect on the health and well-being of the student community need further research. Technology-enabled health promotion initiatives are the emerging trend, including mobile apps, AI, and big data insights, for students’ health promotion.

 However, data on the implementation and post-implementation effectiveness of health promotion initiatives in higher education settings are limited. There is a significant need for further initiatives and research, particularly in oral health, sexual health, and the prevention of non-communicable diseases. Moreover, cost-effectiveness analyses of policy frameworks and systematic outcome assessments of interventions targeting mental and physical health are warranted. Advancing this agenda requires rigorous, interdisciplinary research focused on developing affordable and scalable technologies for campus health promotion. The active participation of LMICs, especially those in Sub-Saharan Africa, is essential to ensure global relevance and equity in higher education health promotion strategies. It is also recommended that health promotion be integrated as a compulsory component of the academic curriculum across institutions. Embedding health promotion indicators into the accreditation and ranking criteria of higher education institutions could significantly elevate their status and prioritisation. Additionally, the development of regional networks and policy frameworks is vital to monitor progress, facilitate inter-institutional learning, and provide strategic guidance for campus-level implementation.

## Competing Interests

 There are no competing interests related to this project.

## Ethical Approval

 Ethics committee approval is not required as it is a review used only secondary data.
